# Foetal Exposure to Maternal Passive Smoking Is Associated with Childhood Asthma, Allergic Rhinitis, and Eczema

**DOI:** 10.1100/2012/542983

**Published:** 2012-08-13

**Authors:** S. L. Lee, T. H. Lam, T. H. Leung, W. H. S. Wong, M. Schooling, G. M. Leung, Y. L. Lau

**Affiliations:** ^1^Department of Paediatrics & Adolescent Medicine, Li Ka Shing Faculty of Medicine, The University of Hong Kong, Hong Kong; ^2^School of Public Health, The University of Hong Kong, Hong Kong; ^3^Department of Health, The Government of The Hong Kong Special Administrative Region, Hong Kong

## Abstract

*Objective*. We examined the hypothesis that foetal exposure to maternal passive smoking is associated with childhood asthma, allergic rhinitis, and eczema. *Methods*. The study was a population-based cross-sectional survey of Hong Kong Chinese children aged ≤14 years carried out in 2005 to 2006. *Results*. Foetal exposure to maternal passive smoking was significantly associated with wheeze ever (OR 2.05; 95% CI 1.58–2.67), current wheeze (OR 2.06; 95% CI 1.48–2.86), allergic rhinitis ever (OR 1.22; 95% CI 1.09–1.37), and eczema ever (OR 1.61; 95% CI 1.38–1.87). Foetal exposure to maternal active smoking was significantly associated with asthma ever (OR 2.10; 95% CI 1.14–3.84), wheeze ever (OR 2.46; 95% CI 1.27–4.78), and current wheeze (OR 2.74; 95% CI 1.24–6.01) but not with allergic rhinitis ever (OR 1.01; 95% CI 0.70–1.46) or eczema ever (OR 1.38; 95% CI 0.87–2.18). The dose response relationship between wheeze ever and current wheeze with increasing exposure, from no exposure to maternal passive smoking and then to maternal active smoking, further supports causality. *Conclusion*. There is significant association between foetal exposure to maternal passive smoking and maternal active smoking with childhood asthma and related atopic illnesses. Further studies are warranted to explore the potential causal relationship.

## 1. Introduction 

Second-hand smoke (SHS) exposure is one of the most important global public health hazards. There have been extensive convincing studies on the adverse health effects of SHS on both adults and children [1, 2]. Prenatal maternal active smoking has been considered as foetal passive smoking. The link between foetal exposure to maternal active smoking with lower birth weight increased risk for sudden infant death syndrome, reduced lung function, and increased respiratory tract infections, and asthma has been well established [1]. The negative effect of prenatal tobacco exposure on the foetus has also been observed for maternal passive smoking in nonsmoker. Previous studies showed that infants born to nonsmoking women exposed to SHS during pregnancy had lower birth weight compared to the nonexposed group [1]. A recent study suggested an effect of foetal exposure to maternal passive smoking on current wheeze although the effect was only observed in those exposed in the third trimester of pregnancy [3]. In Hong Kong, a population health survey showed that the prevalence of daily smoking in adults was 14.7% and there were far greater more male than female smoked (26.8% versus 4.7%) [4]. However, the exposure to second-hand smoke during pregnancy was reported to be 65% in nonsmoking mothers [5]. A birth cohort study [5–7] showed that SHS exposure during pregnancy in nonsmoking mothers was associated with higher medical consultation and hospitalization in their children, and the effect extended beyond early childhood [8]. In addition, there is an apparent increasing trend of childhood asthma, allergic rhinitis, and eczema not just worldwide also in Hong Kong in recent decades [6]. We therefore conducted a study to examine the hypothesis that foetal exposure to maternal passive smoking is associated with childhood asthma, allergic rhinitis, and eczema. 

## 2. Methods

### 2.1. Sampling Strategy and Sample Size

We collected the data via a population-based cross-sectional in-person household health survey of Chinese children aged ≤14 years in Hong Kong during September 2005 to June 2006. The target sample size was set to be approximately 7,500 subjects. Details of the methods are shown in Child Health Survey Report 2005-2006, Department of Health, Hong Kong (accessed via http://www.chp.gov.hk/en/index.html). In brief, sampling units were selected using stratified replicated sample based on the frame of quarters maintained by the Census and Statistics Department (C&SD) at the first stage. For the second stage, households with children aged ≤14 residing were selected and all children aged ≤14 in the sampled households were included. The survey was approved by the Ethical Committee of Department of Health, Hong Kong. Written consent was obtained from parents. 

### 2.2. Survey Instruments

We obtained information from a parent as proxy respondent when the child was aged under 11, and both directly from the child and from a parent for those aged 11 to 14. We conducted face-to-face interview to obtain household, family and personal characteristics, general health information, prenatal maternal passive smoking and active smoking, and household exposure to ETS at the time of survey. Respondents also completed a Chinese version of International Study of Asthma and Allergies in Childhood (ISAAC) questionnaire [9, 10]. 

### 2.3. Foetal Exposure to Maternal Smoking

We used a structured questionnaire [5, 7] to assess foetal exposure to maternal smoking. We ascertained maternal passive smoking by the positive response to the question “Was the mother exposed to second-hand smoking during pregnancy where second-hand smoking (SHS) refers to the exposure to smoke from a lighted cigarette from someone nearby or the smoke blown out by a smoker?” This included SHS from any sources. We counted participants who replied “don't know” or refused to answer as no exposure to maternal passive smoking during pregnancy. We documented maternal active smoking by the question “Did the mother smoke during the pregnancy?” We defined maternal active smoking during pregnancy as mother reported smoking at any stage and at any frequency during the indexed pregnancy. We counted those who replied “don't know” or refused to answer as no maternal active smoking. We then categorized foetal exposure level into 3 levels—no exposure that is, no maternal passive or active smoking; maternal passive smoking only; maternal active smoking irrespective of presence or absence of maternal passive smoking. We also collected information of smoking status of each participant's parents and household members at the time of survey.

### 2.4. Health Outcomes

We selected 5 health incomes of interest which included asthma ever, wheeze ever, current wheeze, allergic rhinitis ever, and eczema ever from the ISAAC questionnaire. They were defined by positive response to the questions “Has your child *ever* had asthma?”, “Has your child *ever* had wheezing or whistling in the chest at any time in the past?” “Has your child had wheezing or whistling in the chest in the *last 12 months*?” “Has your child *ever* had allergic rhinitis?”, and “Has your child *ever* had eczema?”, respectively. We merged missing answers with negative answers according to instructions from ISAAC. 

### 2.5. Potential Confounders

We extracted information on participants and their parents' characteristics, perinatal factors, and personal and family history of atopic illnesses from the structured questionnaire. We chose potential confounders based on previous studies which included socioeconomic status (SES) as defined by the highest education level of either parents (no or primary level, secondary level, tertiary, or above), mode of delivery (vaginal including missing answers versus caesarean section), perinatal problem (presence of any complications in the perinatal period), respondent of interview (father or mother), birthplace (Hong Kong or outside Hong Kong), main child care-giver (father, mother, or other), current parental smoking (yes if either or both parents smoke at the time of survey), and total number of household smokers including the parents (0, 1, ≥2). As there were significant missing responses in questions about gestational age at birth and birth weight, and both could be affected by maternal active and passive smoking [1] we did not include these factors in the analysis. 

### 2.6. Statistical Methods and Analysis

We used descriptive statistics of proportions and means to describe the sample characteristics. We estimated the associations between baseline characteristics of the participants with foetal exposure to maternal smoking and health outcomes using exact chi-square test and selected those characteristics that were associated with both exposure and outcome with a *P* value <0.05 as confounders. We used Cochran-Mantel-Haenszel test to assess possible trend in the levels of exposure with health outcomes if associations were detected. Two models of logistic regression analysis were performed with adjustment of confounders. All confounders in addition to age and sex were included in Model 1 while only age and sex were adjusted in Model 2 to avoid overcontrol of factors that might lie in the causal pathway between foetal exposure to maternal smoking and the 5 health outcomes [11]. Good internal consistency of questionnaire was documented by comparing the response to diagnosis of asthma based on face-to-face interview and ISAAC questionnaire using weighted kappa method (kappa 0.825, *P* < 0.001). All the association results were presented as odds ratio OR with 95% confidence intervals. All analyses were conducted using SAS Software, Version 9.1. (Cary, NC, USA). *P* values of <0.05 were considered as significant.

## 3. Results

Among the 26,373 valid households with domestic household residing, 19,432 (73.3%) were successfully enumerated, 4,570 (17.3%) refused to participate and 2,461 (9.3%) could not be reached despite multiple attempts of contact. For households successfully enumerated, 4975 were found to have children aged ≤14 residing. A total of 7393 children (3839 boys with mean age 8.7 ± 4.0, median age 9.33 years and 3554 girls with mean age 8.7 ± 4.1, median age 9.25 years) finally completed the survey, representing 884,300 children of the target population. The sample population closely matched that of the C&SD statistics of the population at the time of survey except that there was a slight overrepresentation of male participants, of participants residing in public housing estates and the New Territories—a rural part of Hong Kong but with many new towns developing in recent decades. As a fairly representative sample of the target population was surveyed, the result could be generalized to all children aged ≤14 in Hong Kong. 

The baseline characteristics of the 7393 participants are shown in Table 1. The prevalence (95% confidence interval) of asthma ever, wheeze ever, current wheeze, allergic rhinitis ever, and eczema ever were 3.9% (3.5%–4.4%), 3.3% (2.9%–3.7%), 2.08% (1.8%–2.4%), 23.5% (22.5%–24.4%), and 11.4% (10.7%–12.1%), respectively. 

Five thousand one hundred and nine mothers (69.1%) reported that they never smoked nor were exposed to passive smoking during pregnancy, 2116 (28.6%) reported exposure to passive smoking during pregnancy, and 168 (2.3%) smoked during pregnancy. There were some statistically significant differences when comparing the baseline characteristics across the nonexposure group, the group exposed to maternal passive smoking and the group with maternal active smoking during pregnancy. The proportions of participants with family history of asthma (2.4% versus 2.8% versus 9.5%), parent smoked during time of survey (18.4% versus 43.2% versus 81%), and of age group 0-1 year (7.3% versus 7.1% versus 13%) were significantly the highest in the last group (Table 2). Perinatal and postnatal problem was most frequently reported in the group with maternal passive smoking during pregnancy (17.5%), followed by the group with maternal active smoking during pregnancy (14.9%), and the nonexposure group (12.7%). The proportion of high socioeconomic status as defined by either parent attained tertiary level of education was the highest in the nonexposed group (18.6%), followed by the group with maternal passive smoking (15.6%), and the group with maternal active smoking (6% for the group). Among the baseline characteristics selected for further analysis, presence of family history, presence of perinatal problem, and high SES were associated with all the 5 health outcomes (Table 3). The proportion of participants with asthma ever and allergic rhinitis ever increased with age. Presence of current parent smokers was significantly associated with wheeze ever and current wheeze. There was a borderline significant association of current parent smokers with asthma ever. We have excluded current parents smoked in logistic regression model to avoid over adjustment. 

Table 4 showed that foetal exposure to maternal passive smoking was significantly associated with wheeze ever (OR 2.05; 95% CI 1.58–1.67), current wheeze (OR 2.06; 95% CI 1.48–2.86), allergic rhinitis ever (OR 1.22; 95% CI 1.09–1.37), and eczema ever (OR 1.61; 95% CI 1.38–1.87) and marginally significantly associated with asthma ever (OR 1.28; 95% CI 0.99–1.65). Foetal exposure to maternal active smoking was significantly associated with asthma ever (OR 2.10; 95% CI 1.14–3.84), wheeze ever (OR 2.46; 95% CI 1.46–4.78), and current wheeze (OR 2.74; 95% CI 1.24–6.01) but not with allergic rhinitis ever (OR 1.01; 95% CI 0.70–1.46) or eczema ever (OR 1.38; 95% CI 0.87–2.18). Significant dose response relationships were observed from no exposure to foetal exposure to maternal passive smoking and then active and each of the 5 health outcomes (unadjusted *P* for trend <0.001 for asthma ever, allergic rhinitis ever and <0.0001 for wheeze ever, current wheeze, and eczema ever). The strongest associations were reported between current wheeze with either exposure to maternal passive or active smoking (unadjusted OR 2.06; 95% CI 1.48–2.86 for maternal passive smoking and unadjusted OR 2.74; 95% CI 1.24–6.01 for maternal active smoking). With adjustment of confounders in Model 1 (more confounders) or Model 2 (only age and sex), the results remained similar except for asthma ever. The association for asthma ever became nonsignificant when more confounders were included while it became more significant when only age and sex were adjusted. 

## 4. Discussion

Our study shows a significant relationship between foetal exposure to tobacco smoke via maternal passive smoking and wheeze ever, current wheeze, allergic rhinitis, and eczema. The odds ratio of the associations between the prevalence of wheeze ever and current wheeze with exposure via maternal active smoking is greater than that with exposure via maternal passive smoking suggesting a possible dose response relationship, but further studies are warranted to ascertain it. To the best of our knowledge, our study is the first one to show foetal exposure to tobacco smoke via maternal passive smoking with allergic rhinitis and wheeze ever. 

Maternal smoking is one of the important risk factors associated with adverse respiratory health in children. The effect of SHS exposure during pregnancy in nonsmoking women did not receive too much attention despite sufficient evidence to infer a causal relationship between maternal exposure to SHS during pregnancy and a small reduction in birth weight [1]. Our result adds to the list of the adverse effects of prenatal SHS through nonsmoking mothers to their foetuses. So far, there has been only one study showing positive association of foetal exposure to maternal passive smoking during the third trimester with current wheeze with an OR of 1.42 (95% CI 1.06–1.91) [3]. Our result extended the association to wheeze ever, and our narrow confidence intervals of ORs of both wheeze ever (OR 2.05; 95% CI 1.58–2.67) and current wheeze (OR 1.22; 95% CI 1.09–1.37) with foetal exposure to maternal passive smoking can provide more precise risk estimates. The odds ratio for foetal exposure to maternal active smoking (including SHS exposure as well) in our study with wheeze ever (OR 2.46; 95% CI 1.27–4.78) and current wheeze (OR 2.74; 95% CI 1.24–6.01) were comparable to the respective OR reported by Gilliland et al. [12] (OR 1.8; 95% CI 1.2–2.6 for wheeze ever and OR 2.5; 95% CI 1.4 to 4.4 for current wheeze without cold in children who were born to mothers with active smoking during pregnancy but did not have postnatal exposure to SHS) and Magnusson et al. (OR 1.2; 95% CI 1.1–1.5 for wheeze before 3 years of age) [13]. Our wider confidence intervals however were due to the small number of mothers who smoked during pregnancy. Furthermore, we adjusted for several risk factors of childhood asthma. The result remained similar after adjustment except for asthma ever in Model 1. The presence of current parent smokers was associated with wheeze ever and current wheeze, and borderline associated with asthma ever only and not with the other 3 health outcomes; we did not include current parents smoked in logistic regression model. Even when current parents smoked were included in Model 1, only the associations of foetal exposure to maternal smoking during pregnancy with wheeze ever (OR 2.04; 95% CI 0.93–4.45) and current wheeze (OR 2.10; 95% CI 0.84–5.24) became nonsignificant while the other results remained significant. This could be due to the small sample size of participants with foetal exposure to maternal active smoking. When we avoided overcontrol of these potential confounders by adjusting only age and sex in Model 2, all these results became significant. We suggest future studies to include a larger sample size for stratified analysis or inclusion of more confounders in the model for better delineation of the associations. 

Unlike many cross-sectional studies, the difficulty in ascertaining temporal relationship between exposure and disease outcome is not a major issue in our study. Exposure in our subjects occurred before birth and health outcomes occurred after birth. The dose response relationship between wheeze ever and current wheeze with increasing exposure, from no exposure to maternal passive smoking and then to maternal active smoking, further supports causality. Nevertheless, the association between foetal exposure to maternal passive smoking and asthma ever was only marginally significant, unlike that with wheeze ever. We speculate that the adverse effect of maternal passive smoking or maternal active smoking on foetuses is operated via effects on lung function resulting in wheezing in early childhood, while further progression to asthma will depend on whether there are any other triggers after birth. Further study is warranted to elucidate the underlying mechanisms of maternal passive smoking and maternal active smoking on foetuses although evidence of biological plausibility exists. It is now well understood that the immature detoxification system and immature immune system make a foetus more vulnerable to any environmental toxins. Maternal active smoking during pregnancy lowers uterine blood flow and increases placental vascular resistance. Foetal haemoglobin has a greater affinity for carbon monoxide than adult haemoglobin. The resultant foetal hypoxia and transplacental delivery of many other toxic substances due to maternal smoking may have differential effect on immune and respiratory system during critical periods of in-utero development, leading to different manifestations of allergic diseases, modified further by postnatal environmental effects. More direct evidence supports the hypothesis that maternal SHS exposure, specifically to nicotine, may lead to low birth weight through a pathway of fetal hypoxia [14]. There are also studies that suggest germ-cell mutagenic effect of tobacco smoke on fetuses [15]; thus, the predisposition of developing allergic diseases due to ETS exposure can originate as early as the germ-cell stage. 

The effect of foetal exposure to maternal active smoking on allergic rhinitis and eczema is more controversial when compared to that on asthma and wheezing. While some studies did not find any effect of prenatal passive nor active maternal smoking with development of atopy, eczema, or hay fever [13, 16–18], other studies showed an increased risk of prenatal maternal smoking with sensitization to food allergens [19] and eczema [20]. Apart from the positive association of foetal exposure to maternal passive smoking during the third trimester with current wheeze, Xepapadaki et al. also demonstrated the positive association with eczema ever with an OR of 1.45 (95% CI 1.01–2.08) [3]. However, they failed to document the positive association with allergic rhinitis. It is not surprising as although asthma, allergic rhinitis, and eczema are considered to be allergic in nature, their underlying pathogenesis, genetic susceptibility, and environmental risk factors vary. In fact, the prevalence of asthma ever, wheeze ever, and eczema ever varied notably in our study population, like the result of many other ISAAC studies. Nevertheless, with a sufficiently large sample of nonsmoking mothers exposed to SHS during pregnancy in our population, we are able to show the significant association, albeit small magnitude, between foetal exposure to maternal passive smoking during pregnancy with childhood allergic rhinitis and eczema. We believe that an association did exist between maternal active smoking during pregnancy and childhood allergic rhinitis and eczema, but it failed to reach significance due to the relatively small number of mothers who smoked during pregnancy in our study population only. Additionally, the underrecognized negative impact of foetal exposure to maternal passive smoking in contrast to increasing public education and recognition of ill effect of maternal active smoking resulting in different degree of avoidance during pregnancy in either group of participants could possibly result in underestimates of our OR. On the other hand, it is possible that the ill effect of passive smoking is greater than active smoking not only on the mother but also on their foetuses, given the now well-known fact that some toxins contained in sidestream smoke such as carbon monoxide, ammonia, and benzene, hydrocarbon compounds are present in higher concentration in sidestream than mainstream smoke. 

The prevalence of self-reported maternal active smoking during pregnancy in our study was much lower than Western countries [21, 22]. This could be due to underreporting, but the figure was comparable to a previous local large cohort study [5]. In contrast, maternal passive smoking during pregnancy in nonsmoking mothers was nearly 30%, even higher than the 24% reported in a cross-sectional survey in the United States [23]. This warrants special attention in our locality as a significant proportion of nonsmoking pregnant women can have their foetuses affected via passive smoking and the consequence is clearly detrimental. 

We hereby address the limitations of our study. Firstly, there is potential information bias inherent in a cross-sectional design. It was not likely since our Child Health Survey aimed to explore a much broader scope including general, physical, and psychosocial health status of our children population. Secondly, only a questionnaire was employed to assess exposure and outcome. Large-scale population study and the long latency period between exposure and health outcome of interest precluded the use of personal monitoring or stationary measurement of air nicotine or the use of biomarkers for exposure assessment in our study. Previous studies [5–7] showed fairly good validity of questionnaires to assess SHS exposure. The outcome assessment is of lesser concern as ISAAC questionnaire is a well-validated and widely adopted questionnaire in epidemiological studies. Thirdly, foetal SHS exposure assessment appeared to be crude as we defined maternal active smoking during pregnancy as mother smoking at any stage and at any frequency during the indexed pregnancy, while maternal passive smoking during pregnancy included exposure to second-hand smoking during pregnancy from any sources. Nevertheless, scientific evidence indicates that there is no risk-free level of exposure to SHS and breathing even a little SHS can be harmful [1]. Lastly, misclassification can result from failure of respondents to recall exposure precisely especially for children of older age group. If mothers of children with asthma, allergic rhinitis, or eczema could not recall SHS exposure during pregnancy and were categorized as nonexposed group, it should have biased the association towards null effect. In addition, there has been a substantial literature [24–26] to show the reliability and validity of instruments used for both short-term and long-term maternal recall, up to more than 14 years in one study [24], for the pregnancy of interest. 

To conclude, our study shows the significant association of foetal exposure to maternal passive smoking with wheezing, allergic rhinitis, and eczema. This adds to the list of adverse effects of prenatal ETS through nonsmoking mothers to their foetuses. While most of the aggressive antismoking campaigns during pregnancy are addressed to smoking women [27], our results together with evidence from other studies support that smoking cessation campaigns should target all family and household members and colleagues in workplaces of nonsmoking expecting mothers.

## Figures and Tables

**Table 1:** Baseline characteristics of survey participants (*N* = 7393).

	Number	(%)
Age group, years		
0-1	548	7.41
2–5	1433	19.38
6–10	2755	37.26
11–14	2657	35.94
Sex		
Female	3554	48.07
Male	3839	51.93
Highest education level of parents		
No and primary	660	9.05
Secondary and matriculation	5344	73.29
Tertiary	1288	17.66
Perinatal and postnatal problem		
Yes	1044	14.12
No	6349	85.88
Current parent smoking		
No	5404	73.10
Yes	1989	26.90
Total no. of household smokers		
0	5298	71.66
1	1796	24.29
≥2	299	4.04
Family history of asthma		
Yes	199	2.69
No	7194	97.31
Family history of atopy		
Yes	612	8.28
No	6781	91.72
Respondents		
Father	2127	28.77
Mother	5266	71.23
Birth place		
Hong Kong	6556	88.68
Non-Hong Kong	837	11.32
Mode of delivery		
Normal spontaneous delivery + other vaginal	5869	79.39
Cesarean delivery	1390	18.80
Missing	134	1.81
Current primary carer		
Father	523	7.07
Mother	5755	77.84
Other	1115	15.08

**Table 2:** Baseline characteristics of participants categorized according to level of foetal exposure to maternal smoking during pregnancy.

	Number of participants	Unexposed (mother did not smoke and mother not exposed to SHS during pregnancy)	Mother did not smoke but exposed to SHS during pregnancy	Mother smoked with/without exposure to SHS during pregnancy	*P* value
		*n* = 5109	*n* = 2116	*n* = 168	
Family history of asthma					
Yes	199	123 (2.4%)	60 (2.8%)	16 (9.5%)	<0.0001
Current parent smoking					
Yes	1989	940 (18.4%)	913 (43.2%)	136 (81.0%)	<0.0001
Perinatal and postnatal problem					
Yes	1044	649 (12.7%)	370 (17.5%)	25 (14.9%)	<0.0001
Highest education level of parents					<0.0001
No and primary	660	451 (8.8%)	200 (9.5%)	9 (5.4%)	
Secondary and matriculation	5445	3709 (72.6%)	1587 (75.0%)	149 (88.7%)	
Teritiary	1288	949 (18.6%)	329 (15.6%)	10 (6.0%)	
Age group, year					0.0003
0-1	548	375 (7.3%)	151 (7.1%)	22 (13.0%)	
2–5	1433	952 (18.6%)	436 (20.6%)	45 (26.8%)	
6–10	2755	1891 (37.0%)	804 (38.0%)	60 (37.1%)	
11–14	2657	1891 (37.0%)	725 (34.3%)	41 (24.4%)	
Sex					0.47
Female	3554	2470 (48.4%)	998 (47.2%)	86 (51.2%)	
Male	3839	2639 (51.7%)	1118 (52.8%)	82 (48.8%)	

**Table 3:** Association between baseline characteristics selected for logistic regression analysis and the 5 health outcomes.

	Numbers of participants	Asthma ever (*N* = 288)	Wheeze ever (*N* = 244)	Current wheeze (*N* = 154)	Allergic rhinitis ever (*N* = 1736)	Eczema ever (*N* = 841)
Family history of asthma						
No	7194	Reference	Reference	Reference	Reference	Reference
Yes	199	10.43 (7.41–14.67)	5.29 (3.47–8.07)	6.26 (3.86–10.16)	2.20 (1.65–2.94)	2.87 (2.08–3.97)
Current parent smoking						
No	5404	Reference	Reference	Reference	Reference	Reference
Yes	1989	1.25 (0.97–1.61)	1.42 (1.08–1.86)	1.44 (1.03–2.01)	0.94 (0.83–1.06)	1.01 (0.86–1.18)
Perinatal and postnatal problem						
No	6349	Reference	Reference	Reference	Reference	Reference
Yes	1044	1.64 (1.22–2.19)	2.00 (1.48–2.70)	2.18 (1.51–3.14)	1.77 (1.53–2.04)	2.00 (1.67–2.38)
Highest education level of parents						
No and primary	660	Reference	Reference	Reference	Reference	Reference
Secondary and matriculation	5445	1.50 (0.91–2.48)	2.29 (1.16–4.49)	4.39 (1.39–13.86)	1.18 (0.96–1.44)	1.28 (0.96–1.70)
Teritiary	1288	1.95 (1.13–3.35)	4.03 (2.00–8.13)	7.74 (2.40–25.03)	1.67 (1.33–2.10)	2.27 (1.66–3.10)
Age group, years						
0-1	548	Reference	Reference	Reference	Reference	Reference
2–5	1433	3.52 (1.39–8.92)	1.37 (0.76–2.44)	1.44 (0.73–2.82)	2.81 (1.98–4.00)	0.85 (0.64–1.13)
6–10	2755	4.77 (1.94–11.74)	1.04 (0.59–1.81)	0.88 (0.46–1.71)	4.71 (3.36–6.59)	0.73 (0.56–0.95)
11–14	2657	5.22 (2.13–12.84)	1.36 (0.78–2.36)	0.99 (0.52–1.92)	4.79 (3.42–6.70)	0.64 (0.49–0.84)
Sex						
Female	3554	Reference	Reference	Reference	Reference	Reference
Male	3839	1.29 (1.02–1.64)	1.87 (1.43–2.44)	2.01 (1.43–2.83)	1.55 (1.39–1.73)	1.00 (0.86–1.15)

**Table 4:** Associations between foetal exposure to maternal smoking and 5 health outcomes including asthma ever, wheeze ever, current wheeze, allergic rhinitis ever, and eczema ever.

Level of exposure	Numbers of participants	Asthma ever^#^ (*N* = 288)			Wheeze ever^##^ (*N* = 244)			Current wheeze^##^ (*N* = 154)			Allergic rhinitis ever^#^ (*N* = 1736)			Eczema ever^##^ (*N* = 841)		
		Unadjusted	Model 1	Model 2	Unadjusted	Model 1	Model 2	Unadjusted	Model 1	Model 2	Unadjusted	Model 1	Model 2	Unadjusted	Model 1	Model 2
Un-exposed (mother did not smoke and mother did not expose to ETS during pregnancy)	5109	Reference	Reference	Reference	Reference	Reference	Reference	Reference	Reference	Reference	Reference	Reference	Reference	Reference	Reference	Reference
Mother did not smoke but exposed to ETS during pregnancy	2116	1.28 (0.99–1.65)	1.27 (0.98–1.64)	1.29 (1.00–1.66)	2.05 (1.58–2.67)^∗∗∗^	2.04 (1.56–2.66)^∗∗∗^	2.00 (1.54–2.61)^∗∗∗^	2.06 (1.48–2.86)^∗∗∗^	2.01 (1.44–2.80)^∗∗∗^	2.03 (1.46–2.82)^∗∗∗^	1.22 (1.09–1.37)^∗∗^	1.22 (1.08–1.38)^∗∗^	1.23 (1.10–1.39)^∗∗^	1.61 (1.38–1.87)^∗∗∗^	1.59 (1.36–1.85)^∗∗∗^	1.60 (1.37–1.86)^∗∗∗^
Mother smoked with/without exposed to ETS during pregnancy	168	2.10 (1.14–3.84)^∗^	1.75 (0.91–3.35)	2.32 (1.26–4.26)^∗∗∗^	2.46 (1.27–4.78)^∗∗^	2.34 (1.18–4.64)^∗^	2.61 (1.34–5.09)^∗∗∗^	2.74 (1.24–6.01)^∗∗∗^	2.36 (1.04–5.33)^∗^	2.72 (1.23–6.01)^∗∗∗^	1.01 (0.70–1.46)	1.14 (0.78–1.67)	1.14 (0.78–1.65)	1.38 (0.87–2.18)	1.31 (0.81–2.08)	1.32 (0.83–2.08)

Values are shown as OR (95% CI) ^∗^ *P* < 0.05, ^∗∗^ *P* < 0.01, and ^∗∗∗^ *P* < 0.001.

Unadjusted Cochrane-Mantel-Haenszel test for trend ^#^ *P* < 0.01, ^##^ *P* < 0.0001.

Model 1: adjusted for age group, gender, highest education level of parents, perinatal and postnatal problem, and family history of asthma.

Model 2: adjusted for age group and gender only.

## Abbreviations

CHS: Child Health Survey
CI: Confidence interval
C&SD: Census and Statistics Department
HK: Hong Kong
ISAAC: International Study of Asthma and Allergies in Childhood
OR: Odds ratio
SES: Socioeconomic status
SHS: Second-hand smoke.
